# Work-related musculoskeletal discomfort of dairy farmers and employed workers

**DOI:** 10.1186/1745-6673-7-23

**Published:** 2012-11-15

**Authors:** Christina Lunner Kolstrup

**Affiliations:** 1 Department of Work Science, Business Economics and Environmental Psychology, Swedish University of Agricultural Sciences, P.O. Box 88, SE-230 53, Alnarp, Sweden

**Keywords:** Ergonomic work factors, Work environment, Physical exertion, Dairy farming, Milking, Agriculture, Questionnaire, Rating scale

## Abstract

**Background:**

Dairy farming is physically demanding and associated with a high frequency of musculoskeletal disorders (MSD). This study investigated and compared work-related MSD, ergonomic work factors and physical exertion in farmers and employed farm workers on dairy farms in Sweden.

**Methods:**

The study comprised 66 dairy farmers, and 37 employed dairy farm workers. A modified version of the general Standardised Nordic Questionnaire was used for analyses of perceived MSD in nine different parts of the body. Perceived physical discomfort was assessed using questions concerning ergonomic work factors. A rating scale was used for analyses of perceived physical exertion. Information about participant demographics was also collected. The response rate amounted to 70%.

**Results:**

The most frequently reported MSD in farmers and farm workers were located in the lower back (50% and 43%, respectively) and the shoulders (47% and 43%, respectively). MSD were also frequently reported in the neck (33%) among farmers, and in the hands/wrist (41%) among farm workers. MSD in the elbows (23%) and feet (21%) were significantly more frequently reported by farmers than farm workers (5%). Female farmers and farm workers both reported significantly higher frequencies of MSD in the neck (48% and 56%, respectively) and hands/wrists (44% and 61%, respectively) than their male colleagues (24% and 5%; 10% and 21%, respectively). In addition, female farm workers had significantly higher reported frequencies of MSD in the upper and lower back (39% and 61%, respectively) than their male counterparts (5% and 26%, respectively). Milking was perceived as a weakly to moderately physically demanding work task. Repetitive and monotonous work in dairy houses was the ergonomic work factor most frequently reported as causing physically discomfort among farmers (36%) and farm workers (32%), followed by lifting heavy objects (17% and 27%, respectively). Female workers had significantly more reported discomfort from repetitive and monotonous work than their male counterparts (50% and 16%, respectively).

**Conclusion:**

Despite the technical developments on modern dairy farms, there is still a high prevalence of MSD and discomfort from ergonomic work factors, particularly among female workers.

## Background

Agriculture is one of the three most hazardous sectors in the working world and farmers and farm workers are exposed to a variety of work-related factors, which can affect their safety and health [1-9].

A number of national and international studies have shown that farming is a physically demanding occupation with work tasks that can cause musculoskeletal disorders (MSD) [10-15]. According to the Swedish Work Environment Authority, 70% of reported occupational diseases among people engaged in Swedish farming relate to the musculoskeletal system, compared with 55% for all other occupations [16]. In the European Union, MSD are the most commonly reported work-related health problems, with 23% of European workers reporting that they suffer from aches and pains in the musculoskeletal system [17].

It is well-known that working with dairy farming, milking in particular, is physically demanding, associated with difficult working postures and movements, repetitive and monotonous work tasks, and also associated with injuries caused by slips, trips and falls on slippery floors or barn fittings, which constitutes a risk factor for development of MSD [4,11,18-26].

A study of working postures in different milking systems revealed that milking in tethered systems involved unacceptable working postures during 38% of the working time, compared with 9% in loose-housing systems with parlour milking [27]. Installation of pipeline milking on rail in tethered systems improved the work postures and decreased the frequency of twisted back postures from 29% to 11% [28]. Further improvements in work posture were observed when milking parlours were introduced, as they resulted in dairy farmers working with a straight back for 85% of the time and with their arms under shoulder level for 76% of the time [29].

MSD are common among dairy farmers and farm workers, especially MSD in the lower back, shoulders, hands/wrists and knees [4,11,13,19,30-35]. Results from a longitudinal study of 1,465 dairy farmers active in 1988 and of 686 farmers active in Scania (the most southerly province of Sweden) in 2002 showed that the incidence of MSD had increased slightly, to 83% of males and 90% of females by 2002 [31], compared with 81% and 84%, respectively, in 1988. Kolstrup et al. [4] found that MSD were frequently reported by dairy farm workers (86%), most often in the upper extremities (52%) and the back (60%). Female dairy farm workers reported MSD more frequently in all body parts, especially the shoulders (71%) and wrists/hands (57%), than their male colleagues (36% and 11%, respectively).

Several studies have shown that milking in parlours is physically strenuous for the upper extremities, especially among females [4,20-22,32,36,37]. Work tasks such as udder drying, pre-milking and attaching the teat cups are reported to be the most physically demanding tasks for the hands/wrists during milking in parlour systems, especially among females [20]. However, a study among dairy farmers working with an average herd size of 45 cows in loose-housing systems with parlour milking and milking shifts lasting two hours showed that milking was light work with respect to heart rate, work posture and perceived exertion [38].

Several studies and reviews have found significant relationships between high work demands, low control, low social support, and MSDs in the back [39-43]. Physically demanding work, such as an intensified work load, monotonous and repetitive work tasks, combined with psychosocial factors, such as time pressure, overtime, low control, and low job satisfaction have been found as possibly predisposing for upper extremity disorders among workers in different occupations [40,41,44]. A study by Kolstrup et al. [45] showed that although dairy farm workers reported high frequencies of MSDs in the ‘back’ and in the ‘upper extremities’, and had high work demands, they also had high level of control and support and were satisfied with their work. The result of the study showed no significant risk factors for MSD associated with the psychosocial work environment which suggests the probability that factors in the physical work environment are more likely to lead to MSDs than factors related to the psychosocial work environment.

The majority of the studies performed to date have been conducted among dairy farmers and few among employed dairy farm workers. Thus, the main aim of the present study was to investigate and compare self-perceived work-related MSD, ergonomic work factors and physical exertion in farmers and in employed farm workers on large modern dairy farms in Sweden.

## Materials and methods

### Subjects

This study formed part of a larger interdisciplinary project in veterinary medicine, animal ethology, animal husbandry and work science. The objective of the parent project was to study the effects of dairy herd size, housing and management on animal health and fertility, and some aspects of human work environment and health. In order to participate in the overall research project, farms had to be geographically located in any of the four major Swedish dairy regions (representing the largest number of dairy cows) and have indoor loose-housing systems with cubicles (free stalls), a parlour, rotary or automatic milking system, at least 50 dairy cows per farm, and at least one employed farm worker. Other requirements were that participants worked at least 20 hours per week in the dairy house and that there had been no major rebuilding of the cow house during the preceding 20 months and no plans for immediate major rebuilding.

In Sweden, there is no official register of dairy farms with loose-housing. However, the Swedish Dairy Association was able to provide the addresses of 632 farms with cubicle barns, constituting 7% of all Swedish dairy farms with >50 dairy cows in 2003. A first questionnaire regarding farm conditions was distributed to these farmers by mail and 458 farms responded in December 2003. Based on the data obtained, 166 dairy farms met the inclusion criteria and were willing to participate.

At the start of the study in December 2004, only 113 dairy farms were still participating in the project. A second questionnaire concerning work environment and health was mailed to these farms and, after one reminder, 79 farms replied (response rate 70%). The study thus comprised 41 male and 25 female farmers and 19 employed male and 18 female farm workers on 79 large modern dairy farms in Sweden. The study was carried out in compliance with the Helsinki Declaration. Ethical approval of the Regional Ethical Review Board for studies involving humans was not judged to be applicable. However, the larger interdisciplinary project was approved by the Regional Ethics Committee for animal experiments, Gothenburg, register no. 176-2003. Written informed consent was obtained from the respondent for publication of this report and any accompanying images. In the following, dairy farmers are referred to as farmers and employed dairy farm workers as workers. Non-respondents were asked to state the reason for choosing not to participate. The main reasons for cited were: lack of time (24 farms), sold the farm (two farms) or did not want to participate in the study (eight farms). The study design was cross-sectional, with retrospective elements.

### Methods

The study was based on a questionnaire and a rating scale. A modified version of the general standardised Nordic questionnaire including a picture of the body sites was used for analyses of perceived symptoms of MSD in nine different parts of the body: *Have you during the last 12 months regularly experienced aches, pains and discomfort in the: neck, shoulders, elbows, hands/wrists, upper back, lower back, hips, knees, or feet?* The respondents could answer *Yes* or *No* to each of these nine questions [46]. The original questionnaire was designed to obtain information concerning perceived MSD both 12 months as well as seven days prior to the study and whether or not the subjects had been prevented from working because of eventual MSD. In order to compare the results of this study to other studies with dairy farmers and workers, results from the 12 month prevalence are presented in this paper. One cluster, perceived MSD ‘in any body part’, was added in order to describe if the participants had experienced musculoskeletal discomforts in at least one of the nine different body parts according to Kuorinka et al. [46].

The questionnaire also contained three questions reflecting the respondents’ perceived physical discomfort from ergonomic work factors: *Have you during the last 12 months regularly experienced physical discomfort from: lifting heavy objects, awkward working postures and repetitive and monotonous work?* The respondents could answer yes or no to each of these three questions [47].

The Borg CR-10 Scale [48] was used for analysis of the perceived physical exertion while performing machine milking: *How would you rate the physical exertion during the work situation milking (i.e. the actual milking process)?* The subjects rated the physical exertion on a scale from 0 (none at all) to 10 (extremely strong).

Questions about the participants’ demographic data were also included in the questionnaire, such as employment (farmers and employed workers), gender, age, body weight, height, smoking habits, physical exercise habits, chronic health problems, history of work-related injury, number of hours per week spent working with dairy cows, number of hours per week milking, number of years spent working with dairy cows and number of dairy cows tended.

**Figure 1:**
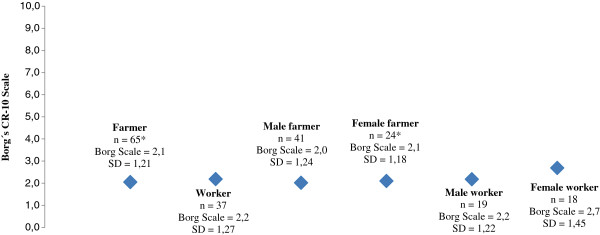
**Perceived physical exertion while performing machine milking.** Descriptive values [number of participants (n), mean value of the Borg CR-10 Scale (Borg Scale) and standard deviation (SD)] are shown according to employment and gender. The Borg CR-10 Scale of perceived physical exertion: 0 = None at all; 0.5 = Extremely weak; 1 = Very weak; 2 = Weak; 3 = Moderate; 5 = Strong; 7 = Very strong; 10 Extremely strong. * One missing value.

The descriptive statistics regarding demographics, perceived work-related musculoskeletal discomfort, physical discomfort from ergonomic work factors and physical exertion during machine milking were illustrated by number (n), frequency (%), mean, median, standard deviation (SD) and statistical significance, and are presented by employment and gender in Table 1, Table 2, Table 3 and Figure 1. For statistical analysis of the results, the Mann–Whitney test and X^2^ analysis (Fisher’s exact test) were used. The probability limits for evaluating statistical significance were: (1) = p < 0.05; (2) = p < 0.01 and (3) = p < 0.001. The IBM SPSS Statistics programme 20 for Windows was used for statistical analysis [49].

**Table 1:** Demographics of dairy farmers and employed dairy farm workers

**Demographics**	**Dairy Farmers**			**Employed Dairy Farm Workers**		
	**Male and female**	**Male**	**Female**	**Male and female**	**Male**	**Female**
Employment ^[a]^	66 (64)			37 (36)		
Gender ^[a]^		41 (62)	25 (38)		19 (51)	18 (49)
Age (years) ^[b]^	47 (10)*	47 (10)*	46 (10)	34 (8)	35 (9)	33 (8)
Height (cm) ^[b]^	175 (9)	179 (6)	167 (7)	178 (10)	186 (6)	170 (7)
Weight (kg) ^[b]^	79 (14)	85 (12)	69 (12)	80 (14)	88 (11)	71 (10)
Smoking (yes) ^[a]^	3 (5)	1 (2)	2 (8)	6 (16) ^**[c1]**^	5 (26) ^**[d2]**^	1 (6)
Taking physical exercise regularly (at least 2 hours per week) (yes) ^[a]^	18 (27)	7 (17)	11 (44) ^**[f1]**^	15 (41)	4 (21)	11 (61) ^**[f1]**^
Having chronic health problem (e.g. diabetes, allergy, asthma), (yes) ^[a]^	3 (5)	1 (2)	2 (8)	8 (22) ^**[c2]**^	2 (11)	6 (33) ^**[e1]**^
History of work-related injury during last 12 months (yes) ^[a]^	6 (9)	5 (12)	1 (4)	5 (14)	1 (5)	4 (22)
Time per week spent working with cows (h) ^[b]^	39 (15)*	40 (17)	36 (12)	34 (12)	35 (12)	34 (12)
Time per week spent milking (h) ^[b]^	19 (12)	19 (12)	20 (11)	17 (8)	15 (8)	19 (8)
Work experience with dairy cattle (years) ^[b]^	22 (11)	25 (12)	19 (9)	12 (8)	14 (9)	10 (7)
Dairy herd size ^[b]^	82 (36–153)	83 (44–153)	80 (36–148)	119 (56–278)	115 (65–278)	123 (56–278)

Descriptive values [number (n), per cent (%), mean and standard deviation (SD)] are listed according to employment and gender.

^[a]^ Number (percentage).

^[b]^ Mean (SD or min; max).

^[c]^ Denotes significant difference between farmers and workers.

^[d]^ Denotes significant difference between males.

^[e]^ Denotes significant difference between females.

^[f]^ Denotes significant difference between males and females within the employment groups (farmers and workers).

^1)^ Denotes significance level p < 0.05; ^2)^ denotes significance level p < 0.01.

* One missing value.

**Table 2:** Prevalence of perceived musculoskeletal discomfort in nine different body parts

**Body part**	**Dairy Farmers**			**Employed Dairy Farm Workers**		
	**Male and female (n = 66)**	**Male (n = 41)**	**Female (n = 25)**	**Male and female (n = 37)**	**Male (n = 19)**	**Female (n = 18)**
	**n (%)**	**n (%)**	**n (%)**	**n (%)**	**n (%)**	**n (%)**
Neck	22 (33)	10 (24)	12 (48) ^[b1]^	11 (30)	1 (5)	10 (56) ^[b2]^
Shoulders	31 (47)	16 (39)	15 (60)	16 (43)	6 (32)	10 (56)
Elbows	15 (23) ^[a1]^	8 (20)	7 (28)	2 (5)	1 (5)	1 (6)
Hands/Wrists	15 (23)	4 (10)	11 (44) ^[b2]^	15 (41)	4 (21)	11 (61) ^[b1]^
Upper back	10 (15)*	5 (12)*	5 (20)	8 (22)	1 (5)	7 (39) ^[b1]^
Lower back	33 (50)	18 (44)	15 (60)	16 (43)	5 (26)	11 (61) ^[b1]^
Hips	8 (12)	6 (15)	2 (8)	3 (8)	0 (0)	3 (17)
Knees	14 (21)	8 (20)	6 (24)	8 (22)	4 (21)	4 (22)
Feet	14 (21) ^[a1]^	6 (15)	8 (32)	2 (5)	0 (0)	2 (11)
‘In any body part’	56 (85)	34 (80)	22 (88)	28 (76)	12 (63)	16 (89)

Descriptive values [number (n), percent (%) and significance] are listed according to employment and gender.

^[a]^ Denotes significant difference between farmers and workers.

^[b]^ Denotes significant difference between males and females within the employment groups (farmers and workers).

^1)^ Denotes significance level p < 0.05; ^2)^ denotes significance level p < 0.001.

* One missing value.

**Table 3:** Perceived physical discomfort from ergonomic work factors

**Ergonomic work factor**	**Dairy Farmers**			**Employed Dairy Farm Workers**		
	**Male and female (n = 66)**	**Male (n = 41)**	**Female (n = 25)**	**Male and female (n = 37)**	**Male (n = 19)**	**Female (n = 18)**
	**n (%)**	**n (%)**	**n (%)**	**n (%)**	**n (%)**	**n (%)**
Lifting heavy objects	11 (17)	7 (17)	4 (16)	10 (27)	3 (16)	7 (39)
Awkward working positions	5 (8)	4 (10)	1 (4)	7 (19)	3 (16)	4 (22)
Repetitive and monotonous work	24 (36)	15 (37)	9 (36)	12 (32)	3 (16)	9 (50) ^[a1]^

Descriptive values [number (n), percent (%) and significance] are listed according to employment and gender.

^[a]^ Denotes significant difference between male and female workers.

^1)^ Denotes significance level p < 0.05.

## Results

### Demographics

The demographics of the participants are presented in Table 1. The study comprised 66 farmers (64%) and 37 workers (36%). The proportion of male and female farmers was 62% and 38%, respectively, and of male and female farm workers 51% and 49%, respectively.

As shown in Table 1, the farmers were older (13 years), worked longer hours per week in the dairy house (5 h per week), spent 2 hours more per week milking the cows, had longer experience of working with dairy farming (10 years) and tended smaller dairy herd herds of mainly Holstein Friesian breed (70 dairy cows per herd) than the workers (115 dairy cows per herd). The workers had a significantly higher frequency of chronic health problems (22%) and work-related injuries (14%) during the previous 12 months prior to the study compared with the farmers (5% and 9%, respectively). There were significantly fewer smokers among the farmers (5%) compared with the workers (16%), with male workers having a significantly higher frequency of smoking (26%) than male farmers (2%). Forty-one percent of the workers and 27% of the farmers regularly took physical exercise for at least 2 hours a week.

In general, the female farmers and workers performed physical exercise significantly more frequently (44% and 61%, respectively) than their male counterparts (17% and 21%, respectively). Furthermore, they had a higher frequency of chronic health problems (8% and 33%, respectively) than the males (2% and 11%, respectively). Specifically, the female workers reported significantly higher frequencies of chronic health problems (33%) compared with the female farmers (8%). Furthermore, the female workers reported the highest frequency of work-related injuries (22%) and the female farmers reported the lowest frequency (4%) (Table 1).

### Musculoskeletal discomfort

Perceived work-related MSD were frequently reported in both employment groups (Table 2). Farmers reported higher frequency of MSD ‘in any body part’ (85%) compared to the dairy workers (76%). Additionally, the female dairy workers had more perceived discomfort in at least one body part compared to their male counterparts (89% and 63%, respectively).

In general, females reported higher frequencies of perceived work-related MSD in seven out of nine different body parts than their male counterparts (Table 2). Female farmers and workers both reported significantly higher frequencies of MSD in the neck (48% and 56%, respectively) and in the hands/wrists (44% and 61%, respectively) than their male colleagues (24% and 5%; 10% and 21%, respectively). In addition, female workers reported significantly higher frequencies of MSD in the upper and lower back (39% and 61%, respectively) than male workers (5% and 26%, respectively).

### Ergonomic work factors

Repetitive and monotonous work in dairy houses was the work factor most frequently reported as causing physical discomfort among farmers (36%) and workers (32%), followed by lifting heavy objects (17% and 27%, respectively) (Table 3). Specifically, the female workers reported the highest frequency of physical discomfort from all three ergonomic work factors (repetitive and monotonous work 50%, lifting heavy objects 39% and awkward working positions 22%) compared with the other groups (male and female farmers, and male workers). Furthermore, a significantly higher frequency of repetitive and monotonous work (50%) was reported by female workers compared with their male colleagues (16%).

### Physical exertion

The respondents were asked to estimate the perceived physical exertion (muscle stress) while performing machine milking on a scale from 0 to 10 according to the Borg CR-10 Scale. As Figure 1 shows, farmers and workers rated the physical exertion almost equal within the range from 2.0 (weak exertion) to 2.7 (moderate exertion). Female workers reported the highest value of physical exertion (2.7) of the four groups, although differences were not significant.

## Discussion

This study sought to investigate and compare self-perceived work-related MSD, ergonomic work factors and physical exertion in farmers and employed farm workers on large modern dairy farms in Sweden.

In recent decades, there has been considerable technical development in dairy farming resulting in new production systems and devices e.g. milking rails in tethering systems, light-weight milking clusters and tubes, automatic cluster removers, loose-housing systems with milking parlours, adjustable floors in the parlours, machines for automatic feeding, manure scrapers and strewing of litter and even automatic milking systems [31,50-56]. These technical developments on dairy farms in recent decades should mean that farmers and workers are exposed to lower levels of physical work load and, consequently, an expected decrease in the prevalence of MSD.

However, high frequencies of reported MSD still seem to be associated with dairy farming. In this study, both farmers and workers reported overall high frequencies of work-related MSD and mainly in the lower back, shoulder, neck, knees, and hand/wrists and discomfort from ergonomic work factors such as repetitive and monotonous work. Comparably high frequencies of MSD have also been found in several national and international studies on dairy farmers and workers [4,10,11,18,19,31].

In two previous studies among Swedish dairy farmers with a comparable average age but mainly working in old-fashioned tethered systems with less technical equipment, the prevalence of MSD assessed by the Nordic Questionnaire were about 81–83% among males and 84–90% among females [31,57]. Further, MSD were mainly located to the lower back (56–57%), shoulders (32–54%) and knees (40–41%) and with some prevalence in neck (23% and 36%), hand/wrists (20%–32%) and hips (18%–35%) [31,57]. The overall prevalence of MSD in the present study among farmers working in loose housing systems with milking parlors, was at a comparable level (80% among male farmers and 88% among female farmers), but lower among the workers (76%) and especially among the males (63%). The results of this study compared to results from the previous studies among Swedish farmers revealed that the prevalence of MSD is almost at the same level, but have decreased in the knees and increased in the shoulders and hands/wrist. A possible explanation could be the change to milking systems with different work tasks (more specialised and monotonous work tasks), work loads (increased number of cows to be milked) and work postures (upright work posture in parlor milking systems). In old-fashioned dairy houses where the cows were kept tethered in stalls, milking was performed in physically demanding postures that involved bending and twisting and often involved carrying heavy milking equipment. Several studies have found that the work load and MSD among farmers and workers in loose-housing systems, where milking is performed in an upright standing position and with stationary milking equipment, are concentrated to the upper extremities [4,19,30,31,36,37]. This indicates that with changed milking systems, work loads and working postures, MSD seem to remain at the same level, but might have shifted from the lower extremities to the upper extremities.

An explanation for the overall high reported prevalence of MSD and discomfort from ergonomic work factors could be that work on large modern farms involves more specialised and monotonous daily work tasks than work on farms with a smaller number of dairy cows [20,21,32,37]. In addition, the farmers and workers might have to increase their work tempo, for example in order to keep pace with the capacity of the milking system or to complete an increased amount of work [58]. The workers in general reported less MSD than the farmers. One explanation could be that the workers, because of their younger age and fewer years in the occupation, had been exposed to MSD related risk factors in the environment to a lesser extent than the farmers. Another possible explanation could be the healthy worker effect [59], i.e. that workers experiencing severe ache, pain or injury had changed occupation due to health issues, while healthy farmers stayed in the occupation. A further explanation could be that the younger generation of workers might not accept aches and pains in their musculoskeletal system to the same extent as farmers, and leave the occupation.

It has been shown that extreme work postures, repetitive and monotonous work and heavy lifting in dairy farming constitute risks for MSD, especially in the hands/wrists [20,22,36,37].

The results also revealed that more females, especially female workers, than males reported symptoms of MSD. The female farmers and workers surveyed here had significantly higher frequencies of MSD in the hands/wrists and the female workers in particular reported significantly more discomfort from repetitive and monotonous work. Previous studies have shown that female industrial and farm workers doing repetitive work and lifting heavy objects report more problems in the musculoskeletal system, especially in the upper extremities, than their male colleagues [32,60-63]. In some studies, this has been attributed mainly to uneven division of responsibility and work tasks between males and females [62,64,65]. However, irrespective of their gender, the farmers and workers surveyed in this study performed almost the same work tasks and worked or milked almost the same number of hours per week. One possible explanation for females reporting higher frequencies of MSD than males is that agricultural equipment and machines are often designed to match the physical requirements and capacities of men [22].

Women’s work capacity is lower on average than that of men regarding, for example, muscular strength and aerobic capacity [24,66]. The work loads for females engaged in certain types of heavy agricultural work are often disproportionate to their physical capacity, as observed on our visits to large dairy farms in Sweden. Additional factors not related to work may also be involved in the prevalence of MSD, such as domestic work and biological and cultural differences. For example, it is more acceptable among females than males to admit to feeling aches and pains [67].

The respondents were asked to rate their physical exertion while performing milking and they reported that the task involved weak to moderate physical exertion. This implies that other work tasks such as manual scraping of manure, handling of feed, strewing of litter (sawdust or straw) and cleaning of the milking parlour and equipment contribute to the high prevalence of perceived MSD reported by the respondents [4].The high prevalence of MSD and reported discomfort from ergonomic work factors found in this study indicates that measures need to be taken in order to reduce the physical work load in dairy farming. This is of particular importance with the increasing herd size on dairy farms and the presumed prolonged time spent on work tasks in dairy houses by farmers and workers. It is advisable to reduce the duration of exposure to heavy work loads and to reorganise the work on these large modern farms, because not all the physically demanding work tasks can be minimised with technical solutions. It is also important to teach those involved to practise correct working postures and techniques in order to avoid MSD. The development of better working routines, such as alternating work tasks, limiting the time spent on working with the same task and resting time in between tasks would also be beneficial in preventing MSD among dairy farmers and workers.

### Methodology

The study design was cross-sectional with a retrospective aspect, which must be recognised when the results are interpreted. Furthermore, the study was based on a small selection of farmers and workers, which is a limitation, and therefore it is possible to draw only broad conclusions. In almost all Swedish mail surveys, the response rate has decreased during recent years and a low response rate also limited the representativeness of the study. However, several of the results found in this study have been confirmed in previous studies comprising a considerably larger number of respondents.

The methods used in this study have been tested for reliability and validity, and discussed in Kuorinka et al. [46] regarding MSD (the Nordic Standardised Questionnaire), in Lundqvist [47] regarding the ergonomic work factors, and in Borg [48] regarding the physical exertion (Borg CR-10 Scale). The main reason for using a previously validated questionnaire and rating scale was to allow comparisons to be made with different studies.

The perceived symptoms of MSD, physical discomfort from ergonomic work factors and physical exertion were measured by self-reporting and a self-administered questionnaire and rating scale. The participants ache, pain and discomfort were not medically diagnosed which might have biased the results with over- or underestimation as a consequence, and this needs to be considered in the interpretation of the results in this study [68,69]. However, the study focused on the respondents’ own perceptions of their work environment factors, aches, pains and discomforts, and this subjective perception must be considered if a correct picture of how work influences respondent health is to be obtained [70]. A preferred research design would be to use a triangulation of methods such as medical examination in combination with the participants’ perception such as questionnaires, rating scales and interviews in order to assess MSD.

## Conclusion

The results showed that high prevalence of MSD and perceived discomfort from ergonomic work factors are still not uncommon among farmers and employed workers on Swedish dairy farms. MSD were mainly reported to be located in the lower back, shoulders, neck, hand/wrists and knees and female dairy farm workers reported the highest frequencies of MSD.

## Competing interests

The author declares that she has no competing interests.

## Author’s contribution

The author planned the study, carried out the data collection and analyses, and wrote the manuscript.
